# Exosomal shuttling of miR-126 in endothelial cells modulates adhesive and migratory abilities of chronic myelogenous leukemia cells

**DOI:** 10.1186/1476-4598-13-169

**Published:** 2014-07-11

**Authors:** Simona Taverna, Valeria Amodeo, Laura Saieva, Antonio Russo, Marco Giallombardo, Giacomo De Leo, Riccardo Alessandro

**Affiliations:** 1 Dipartimento di Biopatologia e Metodologie Biomediche, Sezione di Biologia e Genetica, Università di Palermo, Palermo, Italy; 2 Dipartimento di Discipline Chirurgiche, Oncologiche e Stomatologiche, Sezione di Oncologia Medica, Università di Palermo, Palermo, Italy

**Keywords:** Exosomes, Endothelial cells, Chronic Myelogenous Leukemia Cells, miRNA

## Abstract

**Background:**

Recent findings indicate that exosomes released from cancer cells contain microRNAs (miRNAs) that may be delivered to cells of tumor microenvironment.

**Results:**

To elucidate whether miRNAs secreted from chronic myelogenous leukemia cells (CML) are shuttled into endothelial cells thus affecting their phenotype, we first analysed miRNAs content in LAMA84 exosomes. Among the 124 miRNAs identified in LAMA84 exosomes, we focused our attention on miR-126 which was found to be over-overexpressed in exosomes compared with producing parental cells. Transfection of LAMA84 with Cy3-labelled miR-126 and co-culture of leukemia cells with endothelial cells (EC) confirmed that miR-126 is shuttled into HUVECs. The treatment of HUVECs with LAMA84 exosomes for 24 hours reduced CXCL12 and VCAM1 expression, both at the mRNA and protein level, and negatively modulated LAMA84 motility and cells adhesion. Transfection in HUVECs of miR-126 inhibitor reversed the decrease of CXCL12 and restored the motility and adhesion of LAMA84 cells while the over-expression of miR-126, showed opposite effects.

**Conclusion:**

Our results show that the miR-126 shuttled by exosomes is biologically active in the target cells, and support the hypothesis that exosomal miRNAs have an important role in tumor-endothelial crosstalk occurring in the bone marrow microenvironment, potentially affecting disease progression.

## Background

Chronic myeloid leukemia is a myeloproliferative disorder that originates from a hematopoietic stem cell or a multipotent progenitor. The hallmark of the disease is the presence of the Philadelphia (Ph) chromosome generated from a reciprocal t(9:22) (q34:q11) translocation that creates the BCR-ABL oncogene encoding a chimeric oncoprotein with constitutive tyrosine kinase activity [1]. Several mechanisms are involved in the malignant transformation driven by Bcr–Abl [2] and these altered signalling pathways are ultimately responsible for the increased proliferation, altered cell adhesion, inhibition of apoptosis and enhanced bone marrow angiogenesis observed during the progression of the disease [3]. A number of recent studies have described exosomes as new players in modulating the tumour microenvironment, promoting angiogenesis, tumour development and formation of metastasis [4]. We recently showed that CML cell lines such as LAMA84 and K562 and Imatinib-resistant LAMA84 cells as well as patient’s leukemic blasts, release exosomes that directly affect endothelial cells, thus modulating the process of neovascularization. Specifically, the stimulation of HUVECs with LAMA84 exosomes activates signal transduction pathways leading to the release of IL 8 and the induction, *in vitro* and *in vivo,* of an angiogenic phenotype [5-7]. A topic of particular interest is that exosomes contain miRNAs that can be shuttled to target cells and modulate their behavior. MiRNAs are small (19–22 nucleotides) noncoding RNA molecules that bind to partially complementary 3’ UTR of mRNA causing target degradation or translation inhibition [8]. It has been demonstrated that exosomes released by leukemia cells mediate the crosstalk between leukemia cells and endothelial cells. In particular exosomes released from K562 cells contain miR-210 and miR-92a which enhanced endothelial cell migration and tube formation [9,10].

Using miRNA array and miScript Primer Assay we found that miR-126 was expressed 6 fold greater in LAMA84 exosomes compared with cells. Interestingly, miR-126 has been found to be involved in angiogenesis by targeting sprouty‑related protein with an enabled/VASP homology 1 domain (SPRED1) and phosphoinositide‑3‑kinase regulatory subunit 2 (PIK3R2), known negative regulators of VEGF signaling [11].

Moreover, miR‑126 inhibits both CXCL12 and vascular cell adhesion molecule 1 (VCAM1) expression, which are involved in leukocyte homing in bone marrow and adhesion to ECs respectively [12,13].

CXCL12 is a chemokine that binds specifically to the G-protein coupled receptor, CXCR4. I*n vitro* and *in vivo* studies have clearly demonstrated a key role of CXCR4/CXCL12 interactions in the migration of cells within tissues and, more specifically, in the homing of immune cells in the bone marrow [14]. During CML progression, a modulation of CXCR4/CXCL12 chemotaxis gradient may contribute to the mobilization of leukemic cells into the circulation [15,16].

VCAM1 is a cell-cell adhesion molecules constitutively expressed on endothelial cells in bone marrow (BM) venules; which has been found to play an important role in the homing of Philadelphia positive CD34+ to the BM [17]. Interestingly, previous works have demonstrated that CXCL12 up-regulates VCAM1 adhesive function in myeloma cells and chronic lymphocytic leukemia B cells, and that modulation of this pathway can play important roles in the trafficking and localization of malignant cells to the bone marrow [17,18].

In this study, we show that miR-126 transferred to endothelial cells via LAMA84 exosomes directly targets the 3’ UTR of CXCL12 and VCAM1 mRNA, significantly down-regulating the expression and function of both proteins. This modulation could have important consequences in CML progression.

## Results

### HUVECs internalize LAMA84 exosomes

LAMA84 cells release into the culture medium vesicles that we isolated, purified on a sucrose gradient and characterized as exosomes as previously demonstrated from our group [7].

**Figure 1:**
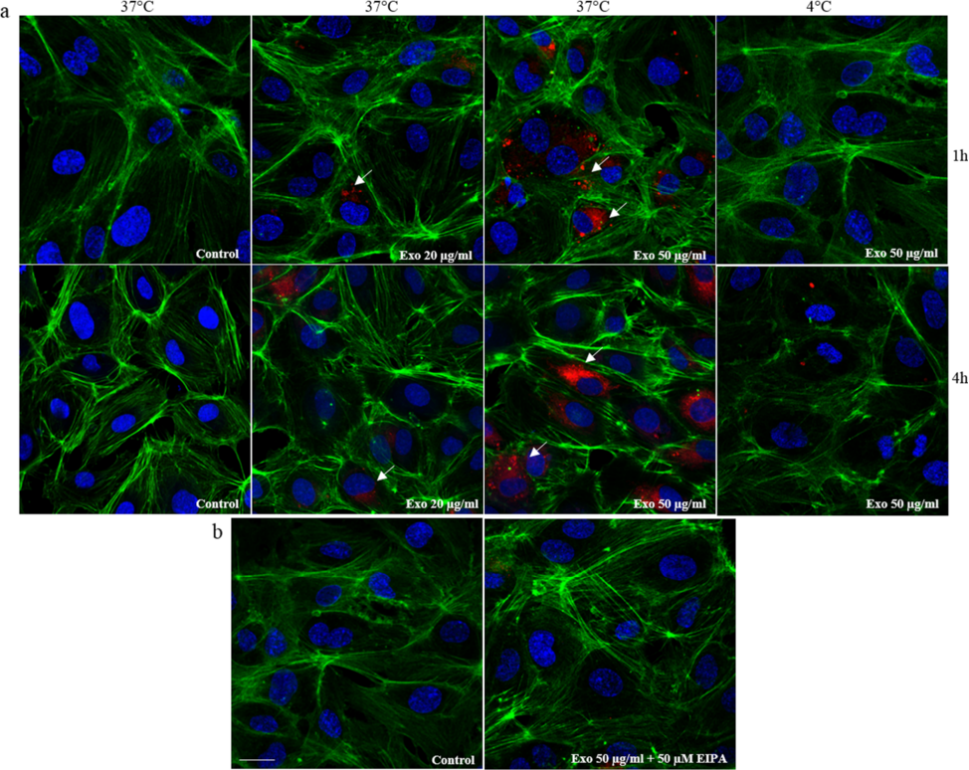
**HUVECs internalize LAMA84 exosomes. a**: Analysis at confocal microscopy of HUVECs treated, for 1 hour and 4 hours, with 20 μg/ml (Exo 20 μg/ml) and 50 μg/ml (Exo 50 μg/ml) of LAMA84 exosomes, compared with untreated HUVECs (Control). HUVECs were stained with phalloidin Alexa Fluor (green), nuclear counterstaining was performed using Hoescht (blue), exosomes were labelled with PKH26 (red). To evaluate whether exosomes uptake was mediated by endocytosis in an energy-dependent process, HUVECs treated with 20 μg/ml (Exo 20 μg/ml) and 50 μg/ml (Exo 50 μg/ml) of LAMA84 exosomes were incubated at 4°C, for 1 hour and 4 hours and compared with untreated HUVECs. **b**: Analysis at confocal microscopy of HUVECs treated, for 1 hour, with 50 μg/ml of exosomes (Exo 50 μg/ml) and EIPA (50 μM), compared with control cells (Control). Scale bar = 10 μm.

The ability of LAMA84 exosomes to be transferred to endothelial cells was studied by examining the uptake of isolated exosomes labeled with PKH-26. HUVECs treated with LAMA84 exosomes, internalized exosomes in a time and dose-dependent manner (Figure 1, panel a). Exosomes rapidly entered into the HUVECs at 37°C and localized in the perinuclear compartment after 4 hours of incubation (Figure 1, panel a). However, the uptake of exosomes in HUVECs was blocked by incubation at 4°C (Figure 1, panel a) or by treatment of endothelial cells with 50 μM EIPA (Figure 1, panel b), a known blocker of macropinocytosis [19] thus confirming that exosomes internalization was mediated by endocytosis in an energy-dependent process, as previously described [12,20]. Semi-quantitative analysis of PKH-26-exosomes fluorescence intensity in the cytoplasm of HUVECs is shown in Additional file 1: Figure S1.

### LAMA84 exosomes transport miRNAs

Analysis with Bioanalyzer of LAMA84 exosomal RNAs showed the presence of abundant short RNAs (data not shown) as previously described [21]. In order to determine the exosomes miRNA profiles and to identify differentially expressed miRNAs in exosomes versus LAMA84 cells, a TaqMan low-density miRNA array was done. We identified 200 miRNAs, among these, 76 miRNAs were only expressed in LAMA84 cells (38%), 18 miRNAs were exclusively expressed in LAMA84 exosomes (9%) and 106 miRNA were differentially expressed between LAMA84 exosomes and LAMA84 cells (53%) (Additional file 2: Figure S2, a). These results showed that LAMA84 exosomes transported 124 miRNAs and suggest a sorting mechanism of miRNAs in exosomes. We used miR-18b for miRNAs normalization it showed no variation between cells and exosomes, though comparable results were obtained using RNU6, a known small nuclear RNA used for miRNAs normalization. We identified 89 miRNAs with increased level (FC > 2) and 17 miRNAs with decreased level (FC < 0.5) in LAMA84 exosomes versus LAMA84 cells (Additional file 2: Figure S2, b). Because we had previously showed that LAMA84 exosomes stimulate *in vitro* and *in vivo* angiogenesis, we focused our attention on angiogenic miRNAs, and particularly on miR-126 which was enriched in LAMA84 exosomes with respect to LAMA84 cells as demonstrated by miRNAs expression profile and single real time quantitative assay (Additional file 2: Figure S2, c).

### Exosome-mediated shuttling of miR-126 into endothelial cells

**Figure 2:**
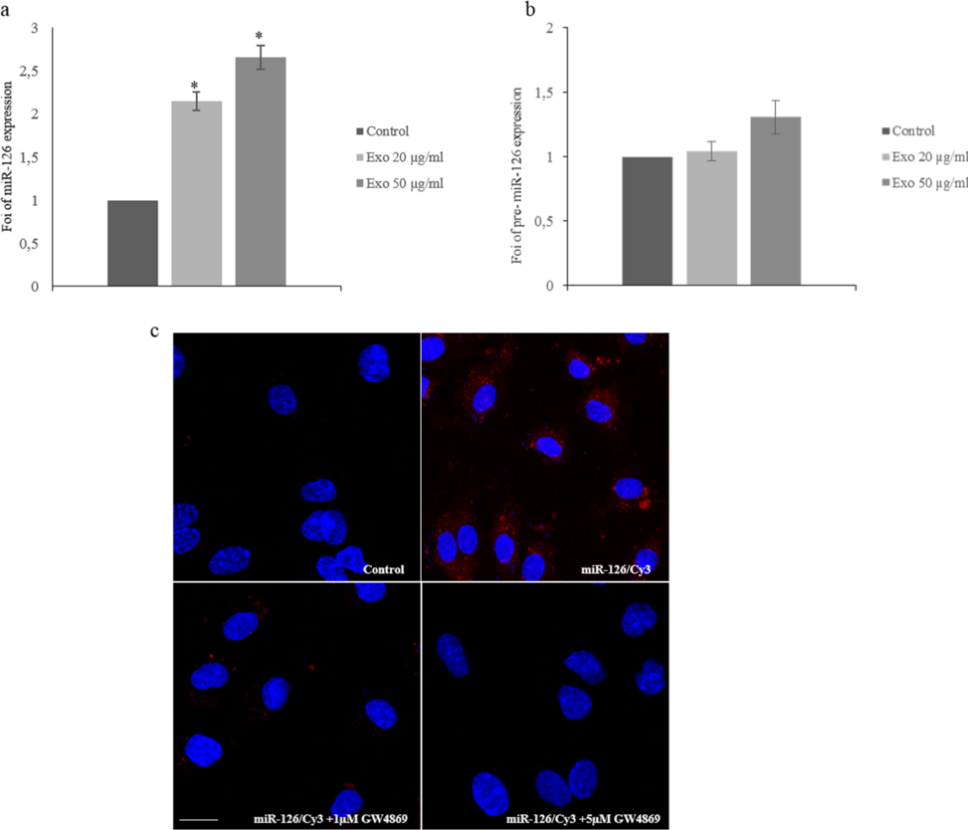
**Exosomes shuttle miR-126 in HUVECs. a**: MiR-126 expression in HUVECs treated with different amounts of LAMA84 exosomes. miR-126 expression levels in HUVECs treated with 20 and 50 μg/ml of LAMA84 exosomes for 24 hours were determined by quantitative Real time PCR analysis. Values are the mean ± SD of 3 independent experiments *p ≤ 0.05. **b**: Pre-miR-126 expression in HUVECs treated with different amounts of LAMA84 exosomes. Pre-miR-126 expression levels in HUVECs treated with 20 and 50 μg/ml of LAMA84 exosomes for 24 hours were determined by quantitative Real time PCR analysis. **c**: Localization of exosomal miR-126 into HUVECs. HUVECs were co-cultured with LAMA84/Cy3-miR-126 cells using Transwells. In Red is shown Cy3-miR-126 in the cytoplasm of HUVECs (miR-126/Cy3), nuclear counterstaining was done with Hoescht (blue). As a negative control, HUVECs were co-cultured with untrasfected LAMA84 (Control). HUVECs were also cocoltured with LAMA84/Cy3-miR-126 cells treated with 1 μM (miR-126/Cy3 + 1 μM GW4869) and 5 μM (miR-126/Cy3 + 5 μM GW4869) of GW4869.

In order to demonstrate the uptake of miR-126 in HUVECs, we treated endothelial cells with 20–50 μg/ml of LAMA84 exosomes and, after 24 h, analyzed the expression levels of miR-126 in HUVECs. As shown in Figure 2a, miR-126 was up-regulated in a dose-dependent manner compared with untreated HUVECs. In order to exclude the possibility that LAMA84 exosomes could induce the expression of endogenous miR-126 in HUVECs, we quantified the levels of precursor miR-126 (pre-miR-126) in HUVECs with Real Time PCR. As shown in Figure 2b, we found no statistically significant difference of pre-miR-126 expression level after treatment of EC with 20–50 μg/ml of exosomes. To visualize the transfer of exosomal miR-126 into HUVECs, LAMA84 cells were transfected with Cy3-labeled pre-miR-126 and co-cultured with HUVECs in transwells. After 24 hours of co-culture, Cy3-miR-126 signals were detected in the cytoplasm of HUVECs (Figure 2 panel c). We did not observe red fluorescence in HUVECs cocoltured with untransfected LAMA84 cells (Figure 2 panel c: Control) or treated with 1–5 μM of GW4869, a specific neutral sphingomyelinase (nSMase) 2 inhibitor also known as an inhibitor of exosome release (Figure 2 panel c) [22]. The semi-quantitative analysis of miR-126-Cy3 fluorescence intensity in the cytoplasm of HUVECs is shown in Additional file 2: Figure S2 d.

### Exosomal miR-126 targets CXCL12 and VCAM 3’-UTR mRNA in HUVECs

**Figure 3:**
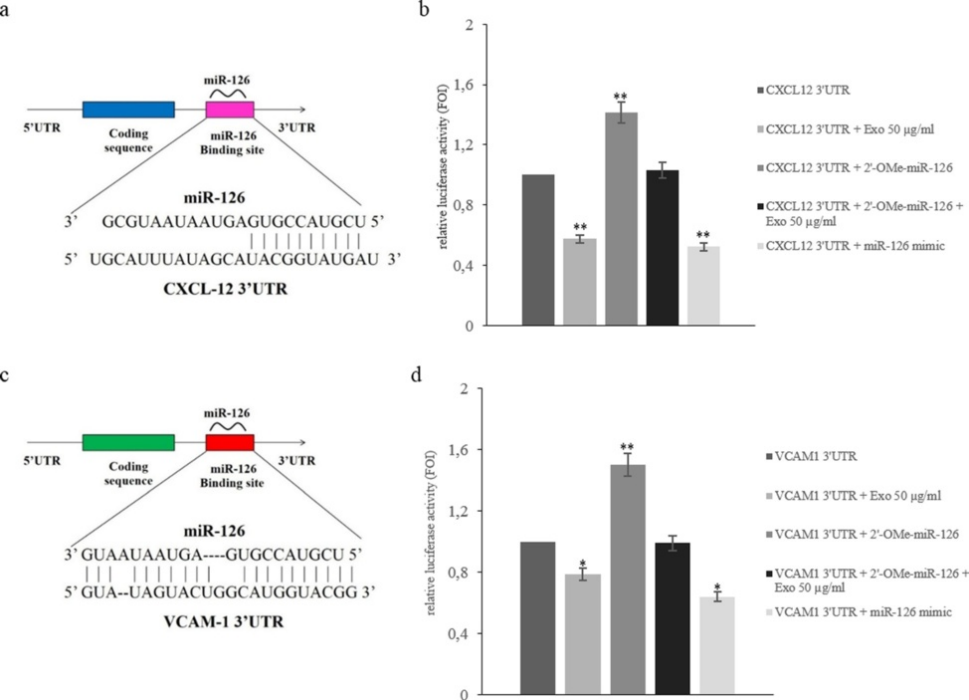
**MiR-126 targets CXCL12 and VCAM1. a**: Schematic representation of matching sequence between CXCL12 3’UTR mRNA and miR-126. **b**: Luciferase activity of HUVECs transfected with reporter plasmid (CXCL12-pEZX), treated with LAMA84 exosomes and/or contrasfected with miR-126 inhibitor or miR-126 mimic. **c**: Schematic representation of matching sequence between VCAM1 3’UTR mRNA and miR-126. **d**: Luciferase activity of HUVECs transfected with reporter plasmid (VCAM1-pEZX), treated with LAMA84 exosomes and/or contrasfected with miR-126 inhibitor or miR-126 mimic. For normalization, Renilla luciferase activity was used. Values are the mean ± SD of 3 independent experiments *p 0.05; **p 0.01.

miRNA target prediction algorithms indicate that CXCL12 and VCAM1 are predictive targets of miR-126. Recent studies report that endothelial miR-126 targets CXCL12 and represses VCAM1 expression in vascular cells [12]. We confirmed that exosomal miR-126 binds to CXCL12 3’UTR mRNA using a Firefly/Renilla Duo-Luciferase reporter vector (pEZX-MT01), where the 3’ UTR of CXCL12 was cloned downstream of the firefly luciferase gene (Figure 3a) (CXCL12-pEZX). When HUVECs transfected with reporter plasmid were incubated with 50 μg/ml of LAMA84 exosomes, the firefly luciferase activity was reduced by 50% compared with untreated HUVECs (Figure 3b) transfected with CXCL12-pEZX. Overexpression of miR-126, by transfection of miR-126 mimic into HUVECs containing CXCL12-pEZX, reduced the activity of firefly luciferase by approximately 45% compared with untransfected HUVECs, similarly to LAMA84 exosomes treatment. In contrast, luciferase activity increased when HUVECs containing CXCL12-pEZX were transfected with miR-126 inhibitor. The treatment of CXCL12-pEZX transfected HUVECs with LAMA84 exosomes, after silencing of miR-126, abolished the increased luciferase activity (Figure 3b). Similar results were obtained when HUVECs were transfected with a Firefly/Renilla Duo-Luciferase reporter vector (pEZX-MT01) containing upstream of luciferase gene the VCAM1 3’UTR fragment (Figure 3c-d). These data indicate that exogenous miR-126 transported via exosomes may function like an endogenous miRNA in HUVECs.

### LAMA84 exosomes modulate CXCL12 expression in endothelial cells

**Figure 4:**
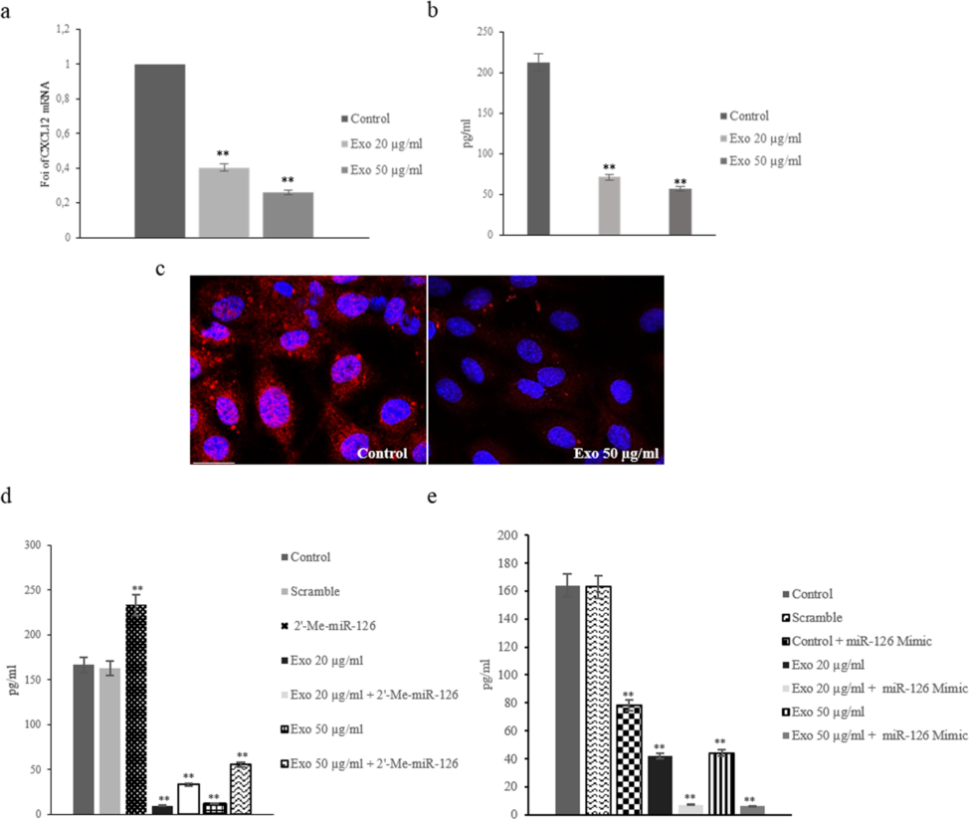
**miR-126 shuttled by exosomes modulate CXCL12 expression in HUVECs. a**: Real time PCR analysis showed that CXCL12 mRNA expression decreased in dose-dependent (20 and 50 μg/ml) manner after addition of exosomes to endothelial cells. Values are the mean ± SD of 3 independent experiments **p ≤ 0.01. **b**: CXCL12 protein levels, assessed by ELISA, in HUVEC-conditioned medium obtained after 24 hours of stimulation with: low serum medium (Control), 20 μg/ml of exosomes (Exo 20 μg/ml) and 50 μg/ml of exosomes (Exo 50 μg/ml). Values are the mean ± SD of 3 independent experiments **p ≤ 0.01. **c**: Confocal microscopy analyses of HUVECs treated with 50 μg/ml of exosomes for 24 h. HUVECs were stained with Texas Red-conjugated anti-CXCL12 antibodies, nuclear counterstaining was performed using Hoescht (blue). Scale bar = 10 μm. **d**: CXCL12 protein levels, assessed by ELISA, in HUVEC-conditioned medium obtained after 24 hours of stimulation with: low serum medium (Control), 20 μg/ml of exosomes (Exo 20 μg/ml) and 50 μg/ml of exosomes (Exo 50 μg/ml). CXCL12 protein levels were also evaluated in HUVECs transfected with miR-126 inhibitor (2-O-Me-miR-126) and treated with: 20 μg/ml of exosomes (Exo 20 μg/ml + 2-Me-miR-126) and 50 μg/ml of exosomes (Exo 50 μg/ml + 2-Me-miR-126). As a negative control, miScript Inhibitor Negative Control (Scramble) was used. **e**: CXCL12 protein levels were also evaluated in HUVECs transfected with miR-126 mimic and treated with: 20 μg/ml of exosomes (Exo 20 μg/ml + miR-126 mimic) and 50 μg/ml of exosomes (Exo 50 μg/ml + miR-126 mimic). As a negative control of transfection, the AllStars Negative Control (Scramble) was used.

We validated the inhibitory effect of miR-126 on CXCL12 expression at both mRNA and protein level. Real time PCR analysis demonstrated that the addition of LAMA84 exosomes to HUVECs, for 24 hours, caused a dose-dependent decrease in CXCL12 mRNA (Figure 4a). CXCL12 mRNA decreased of 60% and 75% in HUVECs treated with 20 and 50 μg/ml of exosomes respectively (Figure 4a). Data were confirmed, at protein level, by ELISA assay; addition of LAMA84 exosomes to HUVECs, for 24 h, caused a dose-dependent decrease in CXCL12 protein in HUVEC conditioned medium (Figure 4b). The results were also confirmed by immunofluorescence assays (Figure 4c).

In order to further assess the function of exosomal miR-126 delivered by LAMA84 exosomes in endothelial cells, we performed an ELISA assay of conditioned medium of HUVECs, transfected with an inhibitor or mimic of miR-126, and then treated with different amounts of exosomes. miR-126 expression was knocked down in HUVECs using the miR-126 inhibitor (2’-OMe-miR-126), as demonstrated with real time PCR assay (Additional file 3: Figure S3a). Inhibition of miR-126 in HUVECs increased the protein level of CXCL12 in conditioned medium and reversed the effects of exosomes treatment (Figure 4d). On the contrary, the ELISA assay indicated that the overexpression of miR-126 in HUVECs significantly decreased CXCL12 protein in conditioned medium and enhanced the effect of exosomes (Figure 4e). Real time PCR analysis showed the overexpression efficiency of miR-126, in HUVECs transfected with miR-126 mimic (Additional file 3: Figure S3b). Our data indicate that the exosomes treatment induces a dose and time-dependent regulation of CXCL12 expression in HUVECs, confirmed by the study of gain and loss of function for miR-126.

### LAMA84 exosomes modulate VCAM1 expression in HUVECs

**Figure 5:**
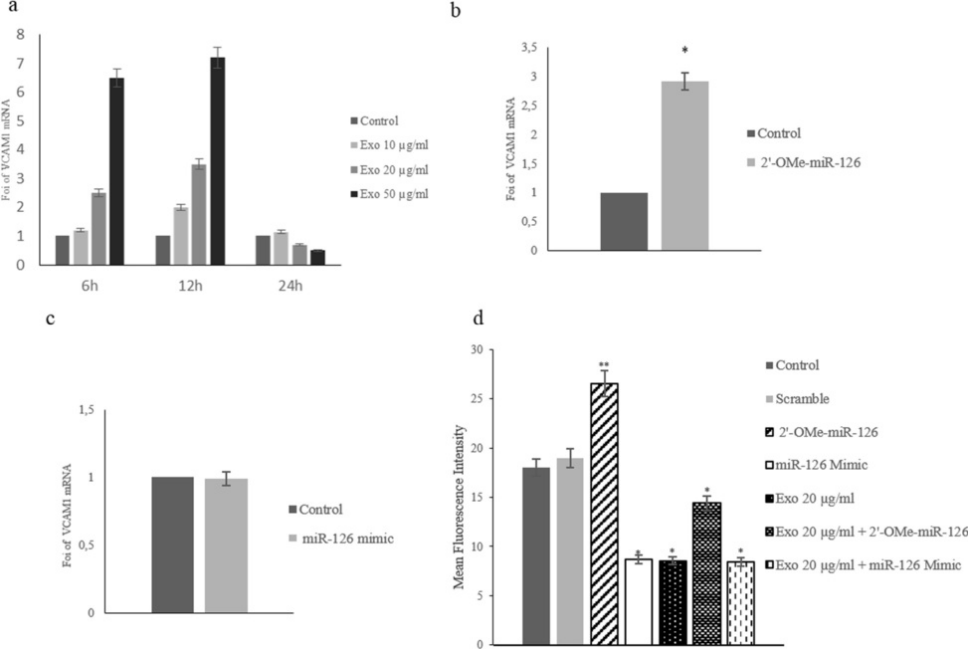
**miR-126 shuttled by exosomes modulates VCAM1 expression in HUVECs. a**: Real Time PCR analysis showed a time dependent modulation of VCAM1 mRNA expression after the addition of 10 (10 μg/ml), 20 (20 μg/ml) and 50 μg/ml (50 μg/ml) exosomes to endothelial cells. **b**: Real Time PCR analysis of VCAM1 mRNA expression levels in HUVECs transfected with miR-126 inhibitor (2-O-Me-miR-126) compared with untrasfected HUVECs (Control). **c**: Real Time PCR analysis of VCAM1 expression levels in HUVECs transfected with miR-126 mimic compared with untrasfected HUVECs (Control).**d**: Histogram shows the MFI (mean fluorescence intensity) of surface expression of VCAM1 in HUVECs after 24 hours of treatment with: low serum medium (Control), 20 μg/ml of exosomes (Exo 20 μg/ml). Surface expression of VCAM1 was evaluated, with FACS analysis, in HUVECs transfected with miR-126 inhibitor (2-Ome-miR-126) and treated with 20 μg/ml of exosomes (2-Ome-miR-126 + Exo 20 μg/ml). As a negative control, miScript Inhibitor Negative Control (scramble) was used. Surface expression of VCAM1 was also evaluated in HUVECs transfected with miR-126 mimic (miR-126 mimic) and treated with 20 μg/ml of exosomes (miR-126 mimic + Exo 20 μg/ml). Values are the mean ± SD of 3 independent experiments *p ≤ 0.05 **p ≤ 0.01.

*In silico* analysis identified VCAM1 3’-UTR mRNA as a target of miR-126; we first evaluated, by real time PCR analysis, if exosomes treatment of HUVECs induces a time-dependent modulation of VCAM1 mRNA expression, As shown in Figure 5a LAMA84 exosomes induce VCAM1 mRNA expression up to 12 h of treatment, in dose dependent manner while there was a decrease in VCAM1 mRNA levels at later time points suggesting a time-dependent regulation of VCAM1 expression. In HUVECs transfected with miR-126 inhibitor (2’-OMe-miR-126), VCAM1 mRNA expression increased 3 fold compared with untrasfected cells (Figure 5b). Forced expression of miR-126 in HUVECs did not affect the relative amount of VCAM1 mRNA (Figure 5c), indicating that VCAM1 mRNA expression was not affected by the increase of miR-126 mimic.FACS analyses indicated that the protein level of VCAM1 expressed in HUVEC surface decreased after 24 hours of treatment with 20 μg/ml of exosomes (Figure 5d) compared with control cells. The inhibition of miR-126 in HUVECs caused an increase of VCAM1 localization on HUVEC surface and reversed the effect of exosomes (Figure 5d). The treatment with LAMA84 exosomes (20 μg/ml) of HUVECs control and transfected with miR-126 mimic did not further affect VCAM1 protein expression compared with HUVECs transfected with miR-126 mimic. It is likely that the small amount of VCAM1 in the HUVEC surface after treatment with exosomes, transporting miR-126, or in HUVECs transfected with miR-126 mimic does not allow observation of a further VCAM1 inhibition. Our data indicate that the exosomes treatment induces a dose and time-dependent regulation of VCAM1 expression in HUVECs, confimed by the study of gain and loss of function for miR-126.

### miR-126 delivered by exosomes reduces LAMA84 cells migration

**Figure 6:**
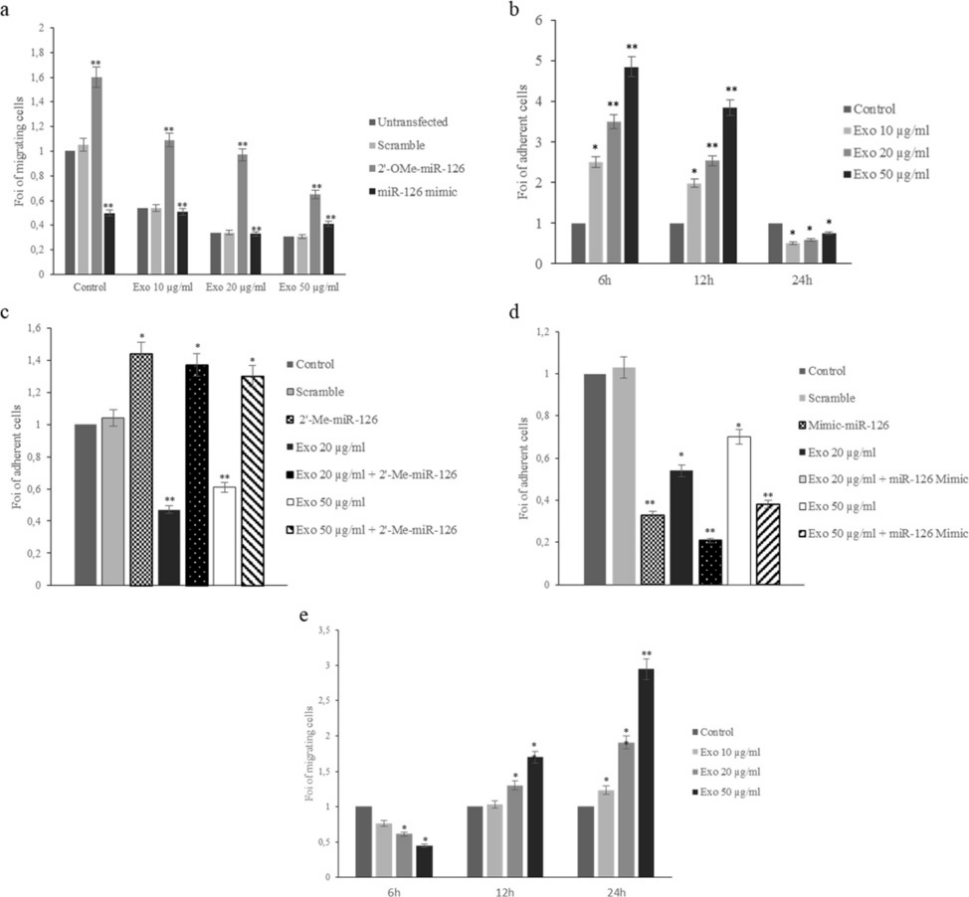
**miR-126 delivered by exosomes modulates LAMA84 migration, adhesion and transendothelial transmigration. a**: Effects of conditioned medium (CM) of HUVECs pretreated with 10 μg/ml 20 μg/ml and 50 μg/ml of exosomes on LAMA84 cells motility compared with CM from untreated HUVECs. LAMA84 cells motility was determined towards conditioned medium of HUVECs transfected with miR-126 inhibitor (2’-O-Me-miR-126) and treated with 10 μg/ml (Exo 10μg/ml, 2’-O-Me-miR-126), 20 μg/ml (Exo 20μg/ml, 2’-O-Me-miR-126) and 50 μg/ml (Exo 50 μg/ml, 2’-O-Me-miR-126) of LAMA84 exosomes compared with untransfected cells. MiScript Inhibitor Negative Control (Scramble) was used as a negative control of miR-126 inhibitor transfection. LAMA84 cells motility towards CM of HUVECs transfected with: miR-126 mimic and treated with 10 μg/ml (Exo 10μg/ml, miR-126 mimic), 20 μg/ml (Exo 20 μg/ml, miR-126 mimic) and 50 μg/ml (Exo 50μg/ml, miR-126 mimic) of LAMA84 exosomes compared with untransfected cells. AllStars Negative Control (Scramble) was used as a negative control of miR-126 mimic transfection. Values are expressed as Fold of Increase (FOI). Values are the mean ± SD of 5 fields in 3 independent experiments *p 0.05; **p 0.01. **b**: Adhesion of LAMA84 cells to HUVECs treated, for 6-12-24 hours, with 20 and 50 μg/ml of LAMA84 exosomes compared with control HUVECs. **c**: Adhesion of LAMA84 cells to HUVECs treated with 20 and 50 μg/ml of LAMA84 exosomes compared with control HUVECs. The adhesion of LAMA84 cells was evaluated on HUVECs transfected with miR-126 mimic and then treated with 20 and 50 μg/ml of LAMA84 exosomes. **d**: The adhesion of LAMA84 cells was evaluated in HUVECs transfected with miR-126 inhibitor and then treated with 20 and 50 μg/ml of LAMA84 exosomes. **e**: HUVECs were grown as a monolayer and treated with 10, 20, 50 μg/ml of LAMA84 exosomes. After 6-12-24 hours of treatment, we evaluated the transendothelial migration of LAMA84 cells.

Cell migration is a critical step for many biologic processes including leukemic blasts mobilization from bone marrow. We analyzed the effects of conditioned medium (CM) from HUVECs treated with exosomes (10–50 μg/ml) on LAMA84 cells motility. Figure 6a shows that LAMA84 cells migration towards HUVEC conditioned medium, for 24 hours, decreased in a dose dependent manner. LAMA84 cells were unable to migrate towards HUVEC conditioned medium in less than 18 hours.When we used conditioned medium from HUVECs overexpressing miR-126 (Figure 6a), we observed a decrease in LAMA84 cells motility, while the silencing of miR-126 in HUVECs increased LAMA84 cells migration compared with untransfected cells (Figure 6a). The use of a CM, from exosome-treated and miR-126 inhibitor-transfected HUVECs, enhanced the LAMA84 cells motility compared with untransfected endothelial cells (Figure 6a). On the contrary, CM from HUVECs, transfected with miR-126 mimic and treated with LAMA84 exosomes, showed a slight modulation of LAMA84 cells motility (Figure 6a), likely because the overexpression of miR-126 could not be further affected by the amount of miRNA shuttled by exosomes. These results indicate that miR-126 transported by LAMA84-exosomes affected LAMA84 cells migration.

### miR-126 shuttled by exosomes modulates LAMA84 cells adhesion on HUVECs

In order to better understand the biological effect of VCAM1 downregulation, we performed adhesion assays. To evaluate if exosomes treatment of HUVECs induce a time-dipendent modulation of LAMA84 cells adhesion on HUVECs monolayer, we performed a time-course adhesion assay. As show in Figure 6b the adhesion of LAMA84 cells on HUVECs monolayer increased up to 12 h compared with control HUVECs while we observed a decreased ability to adhere to endothelial monolayer after 24 h of pretreatment with exosomes.

MiR-126 overexpression in HUVECs decreased the leukemia cell adhesion (Figure 6c) while the silencing of miR-126 in HUVECs reversed the effect of exosomes and restored the adhesion of LAMA84 cells on HUVECs monolayer (Figure 6d). Additional file 4: Figure S4 shows the representative fields used for quantification of LAMA84 cells adhesion on the HUVECs monolayer.

### Effect of miR-126 shuttled by exosomes on transendothelial migration of LAMA84 cells

In order to investigate, in an *in vitro* system, whether the modulation of CXCL12 and VCAM1 expression by exosomal miR-126 might play an active role in leukemic blasts mobilization from the bone marrow, the transendothelial migration assay was performed. HUVECs were grown as a monolayer and treated with different amount (10, 20, 50 μg/ml) of LAMA84 exosomes for 6–12 and 24 hours. As shown in Figure 6e, transendothelial migration of LAMA84 cells toward a complete medium, used as chemoattractant, decreased when HUVECs were treated with exosomes for 6 hours and markedly increased when HUVECs were treated with exosomes for 24 hours.

## Discussion

It is known that in chronic myelogenous leukemia the bone marrow microenvironment contributes to disease progression through the establishment of a bi-directional crosstalk between BM resident cells and cancer cells. This crosstalk may affect therapy response and CML stem cell survival [23]. An important component of bone marrow, in addition to the BM stromal cells (MSC), is represented by endothelial cells; these cells may provide very important cues to tumor development and progression. They form tumor-associated vessels to provide nutritional support to the growing tumor or may sustain leukemia cell growth and dissemination through the secretion of cytokines and extracellular matrix components [24].

Our study adds another piece of information in the complex interaction between the bone marrow microenvironment and cancer cells by introducing the role of cancer secreted microvesicles.

Our previous *in vitro* and *in vivo* work demonstrated that LAMA84 cells release exosomes able to induce in endothelial cells an angiogenic phenotype through the stimulation of an IL-8 dependent autocrine loop [5-7]. In this study we show that LAMA84 cells may modulate *in vitro* gene expression in endothelial cells through the release of miRNAs contained within exosomes. miRNAs profiling evidenced a similarity in the miRNA species found in exosomes and parental cells, however in line with other reports [25] we show that not all miRNAs can be incorporated into exosomes thus suggesting a sorting mechanism, that is still unclear. Our experiments showed in Figure 1 indicate that exosomes, containing miRNAs, are incorporated and released through an energy and ceramide-dependent pathway given that incubation of LAMA84 cells at 4°C or with GW4869 inhibited the transfer of labelled miR-126 into HUVECs. Of the 124 miRNAs we identified in exosomes, we focused on miR-126. miR-126 is considered an angioMiR with an abundant level in highly vascularized tissues, and is known to regulate many aspects of endothelial cell biology including cell motility, vasculature integrity, cell survival and cytoskeletal organization [11]. The involvement of miR-126 in cancer biology is not limited to modulation of angiogenesis and data in literature indicate that this miRNA plays a role in cancers of the gastrointestinal tract, breast, lung and other organs by altering several cellular mechanisms of cancer pathogenesis [26]. In myeloid leukemia miR-126 was found to down-regulate HOXA9, an oncogene encoding a transcription factor that regulates hematopoietic development [27], while Cammarata and colleagues found that miR-126, upregulated in acute myeloid leukemia, induced cell proliferation via the inhibition of PLK, one member of the Polo-like kinase that regulates the cell cycle [28]. Our study suggests a different mechanism by which miR-126 may affect CML development, the alteration of the bone marrow microenvironment due to inappropriate cancer cell retention, adhesion and motility. We provide evidence that CXCL12 and VCAM1, critical components of the bone marrow niche are in part regulated, by miR-126 contained in LAMA84 exosomes. CXCL12 or SDF-1 is a chemokine that binds specifically to the G-protein coupled receptor, CXCR4. Sipkins and colleagues have demonstrated that disruption of the interactions between SDF-1 and its receptor CXCR4 inhibits the homing of Nalm-6 cells (an acute lymphoblastic leukaemia cell line) to the BM [29]. Our data indicate that exosomal delivery of miR-126 to endothelial cells decreases CXCL12 release from HUVECs and concomitantly reduces the motility of LAMA84 cells towards HUVEC conditioned medium. To further study the role of miR-126 in the modulation of CXCL12 secretion, we used the inhibitor of miR-126 that can bind and inhibit miR-126 molecules, or its negative control scramble oligomer, to transfect HUVECs. MiR-126 inhibitor decreased by 45% the miR-126 expression in the cells and concomitantly augmented CXCL12 protein level. Consistently with these data, the overexpression of miR-126 mimic caused a decrease of SDF-1 level and lower migration tendency of LAMA84 cells toward EC conditioned medium. Another target of miR-126 that may be relevant to CML disease progression is VCAM1, a cell-cell adhesion molecule. Experimental studies have demonstrated that miR-126 downregulates VCAM1 expression in endothelial cells through a post-trascriptional mechanism acting on mRNA translation [13]. Fish et al. also reported that VCAM1 mRNA levels were elevated upon miR-126 inhibition, but were not decreased in the presence of miR-126 mimic thus supporting the hypothesis of a regulative mechanism at translational level [12]. Moreover, it was demonstrated that forced expression of miR-126 in the Lin^−^ bone marrow cells induced minimal change in the relative levels of VCAM1 mRNA but caused a decrease in the proportion of surface VCAM1-positive Sca-1^hi^ cKit^hi^ cells within this population [30]. From a functional point of view, a recent report from Salvucci and colleagues reported that miR-126 contained in G-CSF-mobilized vesicles in the bone marrow induced hematopoietic stem/progenitor cell (HSPC) mobilization by reducing the expression of VCAM1 in HSPC endothelial cells and other non-hematopoietic cells [30]. We found that VCAM1 expression is decreased following incubation of endothelial cells with LAMA84 exosomes and that this effect was due to miR-126 contained in the nanovesicles (Figure 5). This effect was partially reduced by the introduction of miR-126 inhibitor in HUVECs (Figure 5). As a functional consequence of the diminished amount of VCAM1 in EC, the adhesion of LAMA84 to HUVECs was reduced after exosome treatment. In our previous study, we demonstrated that the treatment of endothelial cells with exosomes for short times (6 h), induced VCAM1 at both the mRNA and protein level and increased adhesion of LAMA84 cells on the HUVECs monolayer [7].

In this new study, we found that the treatment of endothelial cells with exosomes for 24 hours downregulated VCAM1 mRNA and protein expression and caused a decrease of LAMA84 adhesion cells on the HUVECs monolayer. In order to explain these apparently contrasting results, we hypothesize that in the first 6 hours, the exosomes treatment of HUVECs induces the expression of VCAM1 to allow the adhesion of the cancer cells on the endothelium, as the first step of cells migration. After a longer time of HUVECs exposure to exosomes, LAMA84 cells lose the ability to adhere on the endothelial cells and increase their capacity to migrate towards a richer source of chemoattractants.

The downregulation of CXCL12 and VCAM1 by miR-126 and their upregulation when the miRNAs are knocked down suggests that these miRNA are deeply involved in the regulation of these two proteins. CXCL12 is a chemokine abundantly produced by the bone marrow microenvironment, and its receptor CXCR4 has crucial roles in malignant cell trafficking [31,32]. We demonstrated with an *in vitro* transendothelial cell migration assay that the treatment of HUVECs with LAMA84 exosomes induces LAMA84 migration through endothelial monolayer, likely due to a decrease of VCAM1-mediated adhesion of leukemia cells to EC and a concomitant chemotaxis toward serum.

## Conclusion

Our results indicate a complex exosome-mediated crosstalk potentially occurring in the bone marrow microenvironment that may facilitate the exit of LAMA84 cells from the bone marrow and their dissemination in the body at least partly via delivery of miR-126.

## Material and methods

### Cell culture and reagents

HUVECs were obtained from Lonza (Verviers, Belgium) and grown in endothelial growth medium (EGM, bullet kit, Lonza) according to supplier’s information. LAMA84, (DMSZ Germany) chronic myelogenous leukemia cells, were cultured in RPMI 1640 medium (Euroclone, UK) supplemented with 10% fetal bovine serum (Euroclone, UK), 2 mM L-glutamine, 100 U/ml penicillin and 100 mg/ml streptomycin (Euroclone, UK). All other reagents were purchased from Sigma (St. Louis, MO), if not otherwise cited.

### Exosomes isolation

Exosomes released by LAMA84 cells during a 24 h culture period, were isolated from conditioned culture medium supplemented with 10% FBS (previously ultracentrifuged) by different centrifugations as previously described [7] and isolated vesicles were purified on a 30% sucrose/D2O cushion. Vesicles contained in the sucrose cushion were recovered, washed, ultracentrifuged for 90 min in PBS and collected for use. Exosome protein content was determined by the Bradford method.

### TaqMan Human MicroRNA Array for Profiling of miRNAs

Total cellular RNA and miRNAs were isolated from LAMA84 cells and exosomes using the RNAspin Mini (GE Healthcare Science, Uppsala, Sweden) and analysed through 2100 Bioanalyzer (Agilent Technologies, Santa Clara, CA, USA). 600 ng of total RNA was reverse transcribed using Megaplex™ RT Primers Human Pool A (Life Technologies, Carlsbad, California, U.S.) according to manufacturer’s instructions. Conditions for the reverse transcription reaction were as follows: 16°C for 2 minutes, 42°C for 1 minute, 50°C for 1 second for 40 cycles, 85°C for 5 minutes then hold at 4°C. Obtained cDNA was diluted, mixed with TaqMan Gene Expression Master Mix and loaded into each of the eight fill ports on the TaqMan® Human MicroRNA Array A (Life Technologies, Carlsbad, California, U.S.). The array was centrifuged at 1200 rpm twice for 1 minute each and then run on ABI-PRISM 7900 HT Sequence Detection System (Applied Biosystems, Foster City, CA, USA) using the manufacturer’s recommended program. Data were quantified using the SDS 2.1 software and normalized using the miR-18b as endogenous control or RNU6-2. The cycle threshold (Ct) value, which was calculated relatively to the endogenous control, was used for our analysis (ΔCt). The 2^-ΔΔCT^ (delta-delta-Ct algorithm) method was used to calculate the relative changes in gene expression.

### Quantitative polymerase chain reaction (qPCR) for miRNAs and pre-miRNAs

The expression of miR-126 was validated by miScript PCR System (QIAGEN, Hilden, Germany). Reverse transcription reactions were performed using miScript II RT Kit (QIAGEN, Hilden, Germany) as described by the manufacturer’s instructions. We used miScript HiSpec Buffer for cDNA synthesis to detect mature miRNA and miScript HiFlex Buffer for cDNA synthesis to enable quantification of precursor miRNA, Quantitative Real Time PCR was performed using miScript SYBR Green PCR Kit (QIAGEN, Hilden, Germany). Mature miR-126 was detected by miScript Primer Assay and precursor miR-126 by miScript Precursor Assays according to manufacturer’s instructions. RNU6-2 was used as endogenous control. Expression levels of miRNAs and pre-miRNA were determined using the comparative Ct method to calculate changes in Ct and ultimately fold and percent change. An average Ct value for each RNA was obtained for replicate reactions.

### qPCR for CXCL12 and VCAM1

For CXCL12 and VCAM1 mRNA detection, 1 μg of total RNA were reverse transcribed using the High Capacity cDNA Archive kit (Life Technologies, Carlsbad, California, U.S.), according to manufacturer’s instructions. VCAM1 and CXCL12 transcript levels were measured by TaqMan Real Time PCR using TaqMan gene expression assay for VCAM1 (Hs00174239_m1) and for CXCL12 (Hs00171022_m1) (Life Technologies, Carlsbad, California, U.S.). Data were analysed as previously described [7]. Changes in the target mRNA content relative to GAPDH were determined using the comparative Ct method as described in the previous paragraph.

### HUVECs transfection with miR-126 mimic or inhibitor

Transfection with miScript miR-126 inhibitor (QIAGEN, Hilden, Germany) or miScript miR-126 mimic (QIAGEN, Hilden, Germany) was performed according Fast-Forward Transfection protocol (QIAGEN, Hilden, Germany). 6 × 10^4^ HUVECs per well were seed of a 24-well plate in 500 μl of EGM (Lonza Vervier, Belgium). miR-126 inhibitor (2’-O-Me-miR-126) or miR-126 mimic (2 μM) were diluted in 100 μl culture medium without serum to obtain a final 5 nM miRNA concentration. The cells were transfected using HiPerFect Transfection Reagent (QIAGEN, Hilden, Germany) according to manufacturer’s instructions for 18 hours. miScript Inhibitor Negative Control (QIAGEN, Hilden, Germany) and AllStars Negative Control siRNA (QIAGEN, Hilden, Germany) was used as negative control as indicated by manufacture’s technical specifications. Transfection efficiency was evaluated by quantitative Real Time PCR.

### Luciferase Activity Assay

The 3’-UTRs of CXCL12 and VCAM1 were cloned in pEZX-MT01 vector (Genecopoeia, Rockville, MD USA). The constructs were designed based on the sequence of miR-126 binding sites. 8x 10^4^ HUVECs per well in a 24-well plate were seeded in 500 μl of an appropriate culture medium, the cells were transfected with 300 ng of the pEZX-MT01 firefly luciferase report diluted in 60 μl culture medium without serum. HUVECs were co-trasfected with 6 pmol miR-126 mimic or miR-126 inhibitor using Attractene Transfection Reagent (QIAGEN, Hilden, Germany) according to manufacturer’s protocol. To test whether exosomal miR-126 targets CXCL12 and VCAM1 mRNAs, HUVECs were incubated with 50 μg/ml of LAMA84 exosomes after the transfection with pEZX-MT01 vector. Firefly and Renilla Luciferase activities were measured consecutively using the kit Dual Glo® Luciferase Assay System (Promega Corp., Madison, WI, USA) 24 hours after transfection using GloMax®-Multi Detection System (Promega Corp., Madison, WI, USA). Each transfection was repeated three times in duplicate. Normalized data were calculated as the quotient of Renilla/Firefly Luciferase activities.

### Immunofluorescence analysis

HUVEC monolayers were grown to confluence on coverslips coated with type I collagen (Calbiochem, Darmstadt, Germany) and were treated with 50 μg/ml of LAMA84 exosomes or low serum medium for 24 hours. After incubation, cells were processed as previously described [7]. Antibodies used in the experiments were anti-CXCL12 (1:100; Cell Signaling Technologies). Cells were stained with Texas Red-conjugated secondary anti mouse antibodies (1:100; Molecular Probe, Eugene, OR) and analysed by confocal microscopy (Leica TSC SP8).

### Uptake of LAMA84 exosomes by HUVECs

LAMA84 exosomes were labeled with PKH26 according to supplier’s information. Briefly, exosomes collected after the 100,000 × g ultracentrifugation, were incubated with PKH26 for 10 min at room temperature. Labeled exosomes were washed in PBS by ultracentrifugation, the pellets were resuspended in low serum medium and incubated with HUVECs for 1- 4 hours at 4 or 37°C.

HUVECs were grown on coverslips coated with type I collagen (Calbiochem, Darmstadt, Germany) and were treated with increasing doses of LAMA84 exosomes or low serum medium. In a set of experiments, HUVECs were pretreated with 50 μM 5-ethyl-N-isopropyl amiloride (EIPA), an known inhibitor of exosomes uptake, for 1 hour. After incubation, cells were processed as previously described [7]. HUVECs were stained with Alexa Fluor 633® phalloidin (Molecular Probes, Life Technologies, Carlsbad, California, U.S) that binds F-actin with high affinity. Nuclei were stained with Hoechst (Molecular Probes, Life Technologies, Carlsbad, California, U.S) and analysed by confocal microscopy (Leica TSC SP8). Each picture was acquired with laser intensities and amplifier gains adjusted to avoid pixel saturation. Each fluorophore used was excited independently and sequential detection was performed. Each picture consisted of a z-series of images of 1024–1024 pixel resolution. The semi-quantitative analysis of fluorescence intensity was performed using IMAGE-J software (http://imagej.nih.gov/ij/). We selected the perinuclear area in a section at 5 μm and measured the fluorescence intensity. Values are the mean ± SD of 15 measurements from three independent experiments.

### Shuttling assays for Cy3-labeled-miRNA precursor

miR-126 precursor (QIAGEN, Hilden, Germany) was labeled with Label IT siRNA Tracker Cy3 Kit, according to the manufacturer’s instructions (Mirus, Madison, WI, USA).

LAMA84 cells (6 x 10^4^) were transfected with 10 nM of Cy3-labeled pre-miR-126 using HiPerFect Transfection Reagent (QIAGEN, Hilden, Germany) (LAMA84/Cy3-miR-126). The day after transfection, cells were seeded on transwells, 3 μm pore filters in cocolture with HUVECs over night. LAMA84 cells did not migrate through the 3 μm pore filters through 18 h (data not shown). HUVECs were stained with Hoechst and analysed by confocal microscopy.

### Inhibition of exosome release

LAMA84 cells, transfected with Cy3-labeled pre-miR-126, were seeded in the upper wells of transwells and incubated with 1 and 5 μM GW4869; in the bottom wells were plated endothelial cells. After incubation for 18 hours, the cells were processed as previously described [7] and finally stained with Hoechst and analyzed by confocal microscopy.

### Adhesion assay

Adhesion assays were performed as previously described [33]. Briefly HUVECs transfected or not with miR-126 mimic, miR-126 inhibitor or scramble controls, were grown as a monolayer and incubated for 24 hours with indicated conditions. After treatment, cells were washed with PBS and LAMA84 cells were added for 2 hours at 37°C. Adherent cells were stained with hematoxylin/eosin, each test group was assayed in triplicate; five high power (400x) fields were counted for each condition.

### ELISA for CXCL12

Conditioned medium (CM) of HUVEC, transfected or not with miR-126 mimic or inhibitor, was collected from cells stimulated for 24 hours with different amount of LAMA84 exosomes. CM aliquots were centrifuged to remove cellular debris and CXCL12 protein concentrations were quantified using an ELISA kit (R&D Systems, Minneapolis), according to the manufacturer’s protocol.

### Motility assay

HUVECs transfected or not with miR-126 mimic, miR-126 inhibitor or scramble controls, were grown as a monolayer and incubated for 24 h with different amount of exosomes. After treatment, conditioned medium was collected, CM aliquots were centrifuged to remove cellular debris and used as chemoattractant. LAMA84 cells were suspended in serum-free RPMI 1640 medium supplemented with 0.1% BSA in transwells with 8 μm pore filters and exposed to chemoattractants, for 18 hours. After incubation cells migrated in the bottom wells were counted.

### Transendothelial migration

HUVECs were grown as a monolayer in the upper well of transwells coated with type I collagen, with 8 μm pore filter and incubated with LAMA84 exosomes (10–50 μg/ml). After incubation for 18 hours, LAMA84 cells were added and transmigration of the cells was evaluated after other 18 hours by counting the LAMA84 cells in the bottom well.

### FACS analyses

Expression of HUVEC cell surface VCAM1 was determined by flow cytometry analysis. HUVECs transfected or not with miR-126 mimic or inhibitor were incubated over night with 20 μg/ml of LAMA84-exosomes in a low serum medium (EGM:RPMI, 1:9). 1 x10^6^ cells were washed in PBS and incubated with anti-VCAM1-PE antibody (20 μl) (BD Bioscences, Mountain View, CA, USA) for 15 min at 4°C according to manufacturer’s recommendations. Isotype-matched irrelevant antibodies were used as a negative control. Viable cells were gated by forward and side scatter and analysis was performed on 100,000 acquired events for each sample. Samples were analysed on a FACS Calibur with the use of the CellQuest software (BD Biosciences, NJ, USA).

## Competing interests

The authors declare that they have no competing interests.

## Authors’ contributions

ST participated in the design of the study, performed the isolation of exosomes, the confocal studies and most of the functional assays using the nanovesicle sand drafted the manuscript, VA carried out the microarray studies and luciferase assays, LS participated in the gene expression analysis and in the FACS analysis, AR carried out the statistical analysis and helped in the draft of the manuscript, MG partecipated in the confocal studies and in the migration assays. GDL participated in the design of the study, helped in the statistical analysis and in editing the text. RA conceived the study, and participated in its design and coordination and helped to draft the manuscript. All authors read and approved the final manuscript.

## Supplementary Material

Additional file 1: Figure S1 **a**: Semi-quantitative analysis of PKH-26 fluorescence intensity in the cytoplasm of HUVECs treated with 20 μg/ml and 50 μg/ml of LAMA84-exosomes compared with control cells. HUVECs were incubated at 37°C, for 1 hour and 4 hours. Black bar shows HUVECs treated with 50 μg/ml of exosomes and 50 μM EIPA and incubated at 37°C, for 1 hour. Values are the mean ± SD of 15 measurements from 3 independent experiments *p ≤ 0.05. Click here for file

Additional file 2: Figure S2 LAMA84 exosomes transport miRNAs. **a**: Pie chart representation of 200 miRNAs identified by miRNAs expression profile: 76 miRNAs were only expressed in LAMA84 cells (38%), 18 miRNAs were exclusively expressed in LAMA84 exosomes (9%) and 106 miRNAs were differentially expressed between LAMA84 exosomes and LAMA84 cells (53%). **b**: Heat map analysis showing miRNAs differentially expressed between LAMA84 exosomes and LAMA84 cells. Each row represents the expression levels for a single miRNA tested in LAMA84 cells and LAMA84 exosomes. Each column shows the expression levels for the miRNAs tested in LAMA84 cells (left column) and LAMA84 exosomes (right column). The color scale bar on the top indicates Ct values that correlated to an increase (green) or decrease (red) in the level of miRNA expression. Black boxes indicate intermediate expression values. **c**: miR-126 expression in LAMA84 exosomes and LAMA84 cells. The real time PCR analysis shows that miR-126 is upregulated in LAMA84 exosomes. The data showed in the graph are expressed as FOI (fold of increase) and calculated as 2^-∆∆Ct^. Values are the mean ± SD of 3 independent experiments *p ≤ 0.05. **d**: Semi-quantitative analysis of miR-126/Cy3 fluorescence intensity in the cytoplasm of HUVECs co-cultured with LAMA84/Cy3-miR-126 cells compared to HUVECs co-cultured with untrasfected LAMA84. Values are the mean ± SD of 15 measurements from three independent experiments *p ≤ 0.05. Click here for file

Additional file 3: Figure S3 **a**: Real Time PCR analysis of miR-126 expression levels in HUVECs transfected with miR-126 inhibitor (2-O-Me-miR-126) compared with untrasfected HUVECs (control). Values are the mean ± SD of 3 independent experiments *p ≤ 0.05. **b**: Real Time PCR analysis of miR-126 expression levels in HUVECs transfected with miR-126 mimic (miR-126 mimic) compared to untrasfected HUVECs (control). Values are the mean ± SD of 3 independent experiments **p ≤ 0.01. Click here for file

Additional file 4: Figure S4 Representative fields used for quantification of the LAMA84 cell adhesion. In this figure the LAMA84 cells adhering to HUVECs treated with 50 μg/ml (Exo 50 μg/ml) of LAMA84 exosomes compared with control HUVECs (control) are illustrated. The adhesion of LAMA84 cells was also evaluated in HUVECs: transfected with miR-126 inhibitor (2’-O-Me-miR-126) and HUVEC treated with 50 μg/ml (Exo 50 μg/ml + 2’-O-Me-miR-126) of LAMA84; transfected with miR-126 mimic (mirR-126-Mimic) and treated with 50 μg/ml (Exo 50 μg/ml, miR-126 mimic) of LAMA84 exosomes. Click here for file
