# Supplementary figures and images for: Exosomal shuttling of miR-126 in endothelial cells modulates adhesive and migratory abilities of chronic myelogenous leukemia cells
Source: Mol Cancer. 2014 Jul 11;13:169. doi: 10.1186/1476-4598-13-169 (PMC4105877; doi:10.1186/1476-4598-13-169)

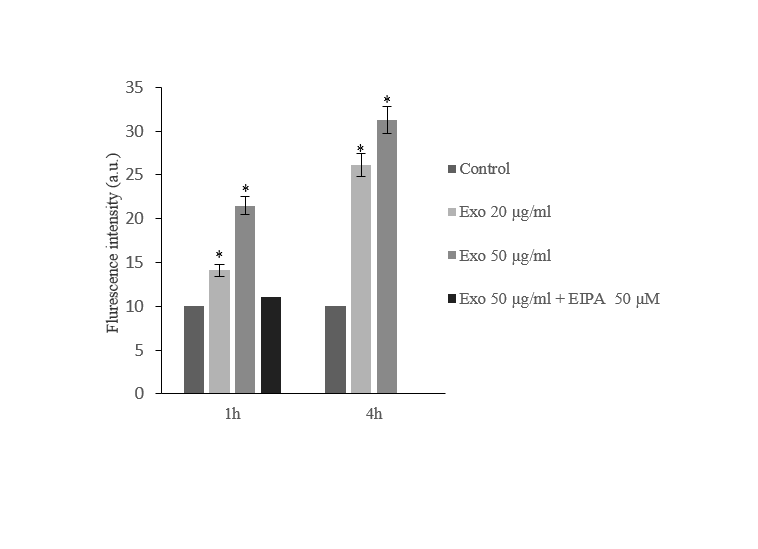

Supplement: Additional file 1: Figure S1 — a: Semi-quantitative analysis of PKH-26 fluorescence intensity in the cytoplasm of HUVECs treated with 20 μg/ml and 50 μg/ml of LAMA84-exosomes compared with control cells. HUVECs were incubated at 37°C, for 1 hour and 4 hours. Black bar shows HUVECs treated with 50 μg/ml of exosomes and 50 μM EIPA and incubated at 37°C, for 1 hour. Values are the mean ± SD of 15 measurements from 3 independent experiments *p ≤ 0.05. [file 1476-4598-13-169-S1.tiff]

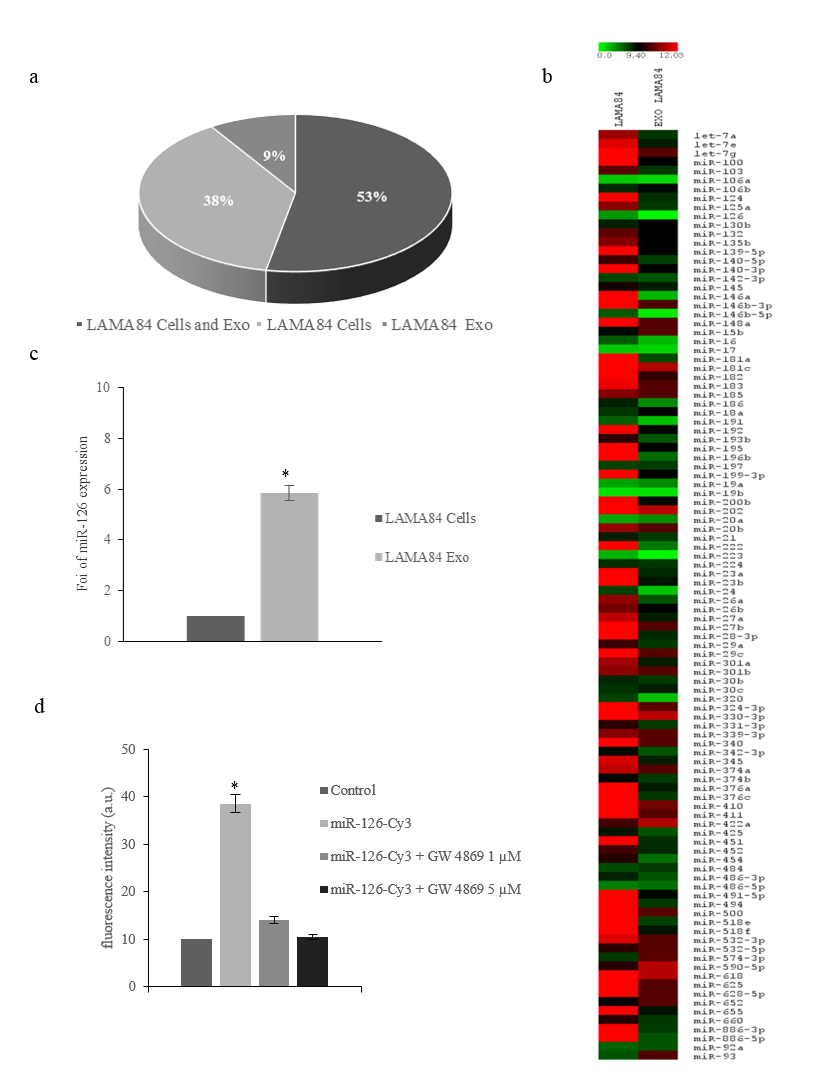

Supplement: Additional file 2: Figure S2 — LAMA84 exosomes transport miRNAs. a: Pie chart representation of 200 miRNAs identified by miRNAs expression profile: 76 miRNAs were only expressed in LAMA84 cells (38%), 18 miRNAs were exclusively expressed in LAMA84 exosomes (9%) and 106 miRNAs were differentially expressed between LAMA84 exosomes and LAMA84 cells (53%). b: Heat map analysis showing miRNAs differentially expressed between LAMA84 exosomes and LAMA84 cells. Each row represents the expression levels for a single miRNA tested in LAMA84 cells and LAMA84 exosomes. Each column shows the expression levels for the miRNAs tested in LAMA84 cells (left column) and LAMA84 exosomes (right column). The color scale bar on the top indicates Ct values that correlated to an increase (green) or decrease (red) in the level of miRNA expression. Black boxes indicate intermediate expression values. c: miR-126 expression in LAMA84 exosomes and LAMA84 cells. The real time PCR analysis shows that miR-126 is upregulated in LAMA84 exosomes. The data showed in the graph are expressed as FOI (fold of increase) and calculated as 2-∆∆Ct. Values are the mean ± SD of 3 independent experiments *p ≤ 0.05. d: Semi-quantitative analysis of miR-126/Cy3 fluorescence intensity in the cytoplasm of HUVECs co-cultured with LAMA84/Cy3-miR-126 cells compared to HUVECs co-cultured with untrasfected LAMA84. Values are the mean ± SD of 15 measurements from three independent experiments *p ≤ 0.05. [file 1476-4598-13-169-S2.tiff]

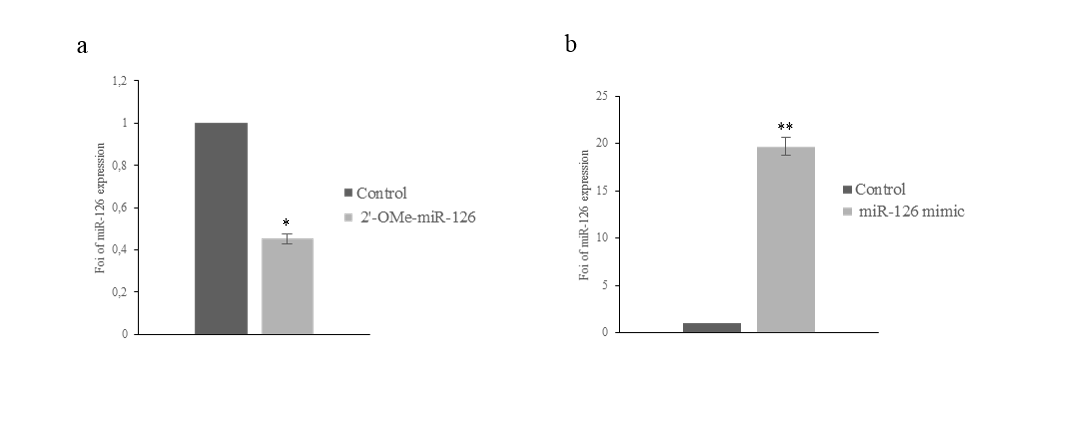

Supplement: Additional file 3: Figure S3 — a: Real Time PCR analysis of miR-126 expression levels in HUVECs transfected with miR-126 inhibitor (2-O-Me-miR-126) compared with untrasfected HUVECs (control). Values are the mean ± SD of 3 independent experiments *p ≤ 0.05. b: Real Time PCR analysis of miR-126 expression levels in HUVECs transfected with miR-126 mimic (miR-126 mimic) compared to untrasfected HUVECs (control). Values are the mean ± SD of 3 independent experiments **p ≤ 0.01. [file 1476-4598-13-169-S3.tiff]

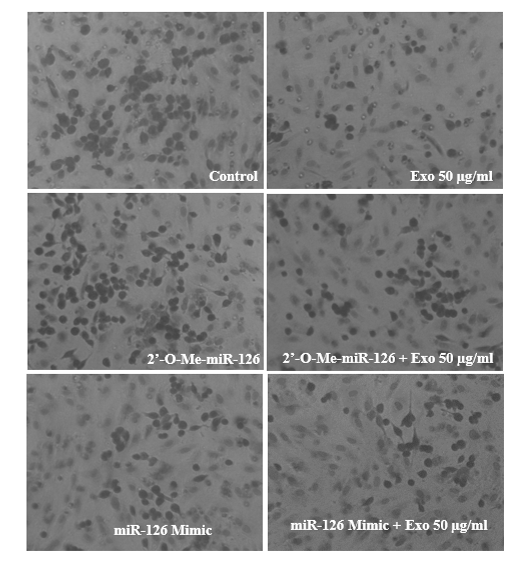

Supplement: Additional file 4: Figure S4 — Representative fields used for quantification of the LAMA84 cell adhesion. In this figure the LAMA84 cells adhering to HUVECs treated with 50 μg/ml (Exo 50 μg/ml) of LAMA84 exosomes compared with control HUVECs (control) are illustrated. The adhesion of LAMA84 cells was also evaluated in HUVECs: transfected with miR-126 inhibitor (2’-O-Me-miR-126) and HUVEC treated with 50 μg/ml (Exo 50 μg/ml + 2’-O-Me-miR-126) of LAMA84; transfected with miR-126 mimic (mirR-126-Mimic) and treated with 50 μg/ml (Exo 50 μg/ml, miR-126 mimic) of LAMA84 exosomes. [file 1476-4598-13-169-S4.tiff]
